# Gene Regulatory Networks from Multifactorial Perturbations Using Graphical Lasso: Application to the DREAM4 Challenge

**DOI:** 10.1371/journal.pone.0014147

**Published:** 2010-12-20

**Authors:** Patricia Menéndez, Yiannis A. I. Kourmpetis, Cajo J. F. ter Braak, Fred A. van Eeuwijk

**Affiliations:** 1 Biometris, Wageningen University, Wageningen, The Netherlands; 2 Centre for BioSystems Genetics, Wageningen, The Netherlands; 3 Laboratory of Bioinformatics, Wageningen University, Wageningen, The Netherlands; Center for Genomic Regulation, Spain

## Abstract

A major challenge in the field of systems biology consists of predicting gene regulatory networks based on different training data. Within the DREAM4 initiative, we took part in the multifactorial sub-challenge that aimed to predict gene regulatory networks of size 100 from training data consisting of steady-state levels obtained after applying multifactorial perturbations to the original *in silico* network.

Due to the static character of the challenge data, we tackled the problem *via* a sparse Gaussian Markov Random Field, which relates network topology with the covariance inverse generated by the gene measurements. As for the computations, we used the Graphical Lasso algorithm which provided a large range of candidate network topologies. The main task was to select the optimal network topology and for that, different model selection criteria were explored. The selected networks were compared with the golden standards and the results ranked using the scoring metrics applied in the challenge, giving a better insight in our submission and the way to improve it.

Our approach provides an easy statistical and computational framework to infer gene regulatory networks that is suitable for large networks, even if the number of the observations (perturbations) is greater than the number of variables (genes).

## Introduction

Traditional methods where one gene or one chemical reaction was studied at a time, have taken step to more sophisticated ones, which try to elucidate the complex machinery connecting all the biochemical reactions happening in a cell. Advanced data collection techniques are able to produce a great variety of data that aim to be the vehicle to better understand the processes within a cell. Development of statistical and mathematical methodology to study such data plays a key role to elucidate and model the mechanisms behind the cell biochemical complex architecture. In particular, it is of great interest to represent the cell biochemistry into networks that mimic the chemical reactions taking place in the cell.

The DREAM project [1], [2], acronym for *Dialogue on Reverse Engineering Assessment and Methods*, is an initiative that tries to motivate the systems biology community to investigate and develop methodologies that translate biochemical processes into gene regulatory networks, by challenging the participants to infer network structure from some given *in silico* gene expression data sets. This *in silico* data were generated by the GeneNetWeaver tool version 2.0 [3] based on the ideas in [4]. The multifactorial sub-challenge, posted in the DREAM4 initiative web page [5] aimed to reverse engineer five gene regulatory networks of size 100 with an experimental scenario assuming that extensive knockout/knockdown or time series experiments, could not be performed. The data for this multifactorial sub-challenge consisted of measurements of steady-state levels of the network, which were obtained by applying 100 multifactorial perturbations to the original network. These steady-state level measurements intrinsically do not give information about the regulatory network dynamics, but about the system equilibrium once it has recovered after the intervention or perturbation.

Given the steady-state nature of the multifactorial sub-challenge data, we focused on Gaussian Markov Random Field theory [6] that leads to the estimation of undirected graphical models [7]. Understanding the topology of a gene regulatory network is equivalent to know which are the connections between the genes involved in the network summarized in the adjacency matrix, that represents the web of connections between the genes of the network.

Gaussian Markov Random Fields theory (GMRF) relates the inverse of the process covariance matrix, described by the elements of the network, in our case a set of genes, with the adjacency matrix that describes the topology of the network. If the 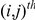 element of the covariance inverse matrix is zero, then variables 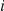 and 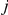 are conditionally independent given the others and do not have an edge in the network. Due to the symmetric nature of inverse covariance matrix the estimated network topology is undirected.

This relation between the covariance inverse and the adjacency matrix links GMRF theory with graphical models, so extending the graphical models provided by relevance networks [8]. The graphical Lasso algorithm [9] is an appealing, new approach to estimate the process covariance inverse and thus appeared very suitable to provide the gene regulatory network under the GMRF umbrella. The graphical Lasso computes the covariance inverse matrix by applying an 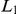 penalty to the GMRF loglikelihood [9], [10], as in the regular lasso [11]. The 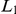 penalty is the sum of the absolute values of the entries of the covariance inverse and due to the geometry of this penalty, the resulting covariance inverse contains entries being exactly zero. The corresponding network is thus sparse. This is an attractive feature of the graphical Lasso, as many of the cell metabolic or enzymatic process networks are known to be sparse [12]. Networks which are very densely connected are unlikely to represent the true biochemical processes within a cell.

## Materials and Methods

### Data sets

**Figure 1:**
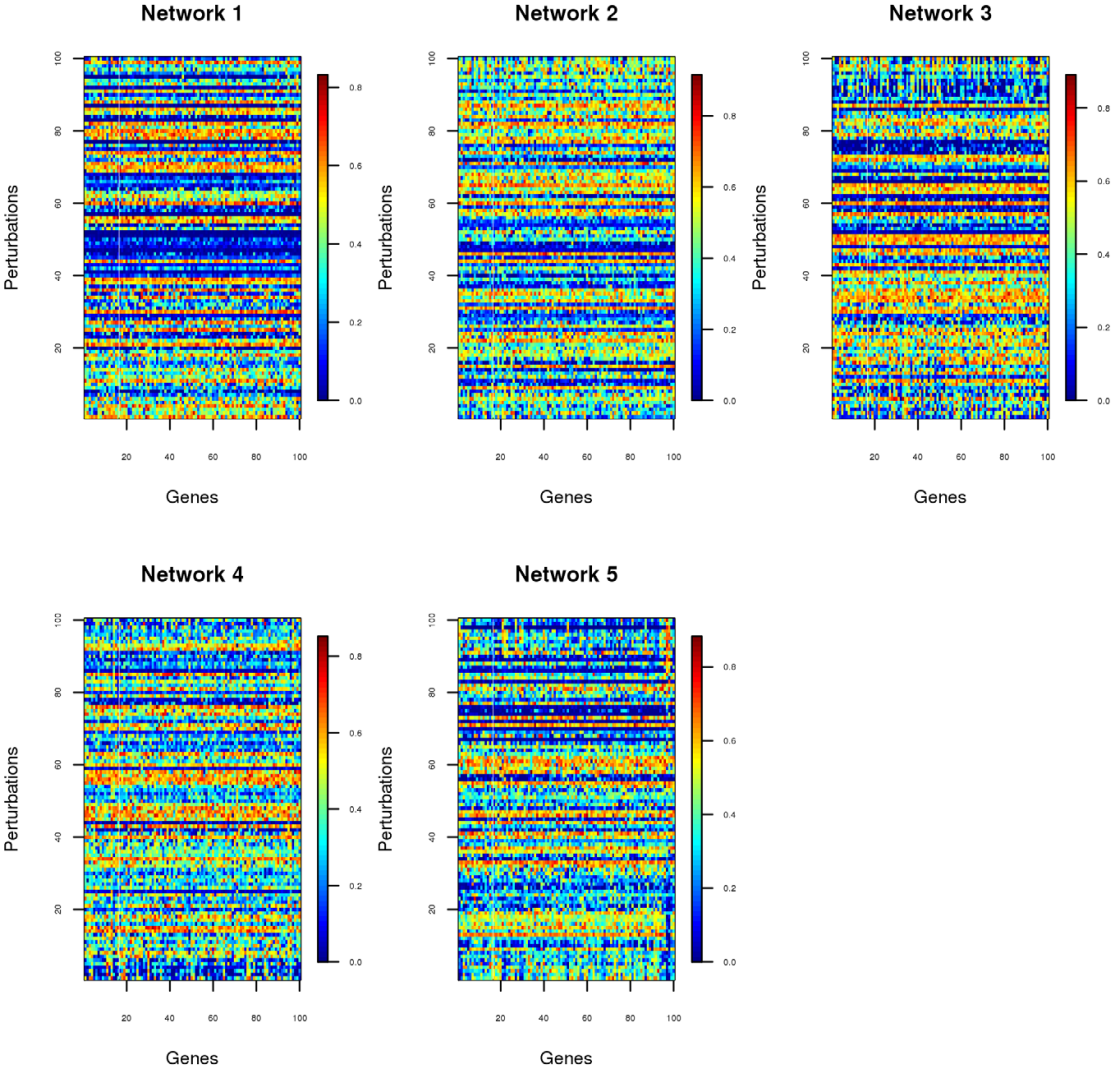
The experimental data. Visualization of the gene levels for all the perturbations ordered according to the first principal component.

The data provided in the multifactorial sub-challenge of DREAM4, consisted of *in silico* networks of gene expression measurements of steady-state levels, obtained by applying 100 different multifactorial perturbations to the original network, containing in total 100 genes. The multifactorial perturbations were induced by slightly increasing or decreasing the basal activation of all the genes in the network simultaneously by different random amounts [5]. If we think of the data in a matrix format, the data set for each network (Fig. 1) consists of a matrix with 100 rows and 100 columns. Each row of this matrix contains the 100 genes expression measurements for the network for a given perturbation, and each column stores the expression levels for a given gene for all the perturbations. From the data matrix we can compute the sample covariance matrix of the gene expression measurements, but this will be a poor estimate of the true covariance matrix 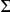 because of the low number of perturbations.

### Gaussian Markov Random Fields (GMRF)

With the aim of predicting the network structures in the multifactorial sub-challenge and considering that the only available data consisted of static records (i.e steady-state levels), it seemed reasonable to tackle the problem by a Gaussian Markov Random Field [6]. A Gaussian Markov Random Field (GMRF) consists of a finite set of random vectors 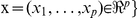 that have a multivariate Gaussian probability density function
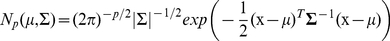
(1)


with mean vector 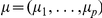 and 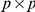 covariance matrix 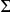. The multivariate Gaussian character of the set 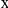 gives the *Gaussian random field* its name. In our case 
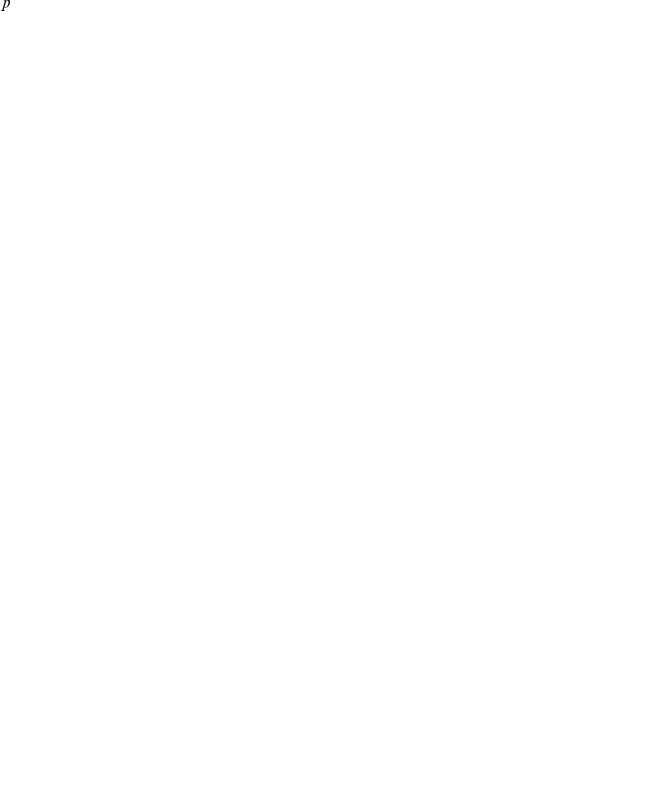
 is the number of genes and 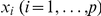 is a random variable representing the *in silico* gene expression measurements for node ( = gene) 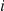 of the network. We consider the rows of the data matrix as a random sample of size 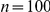 of 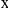.

The *Markov* adjective is for the Markovian global, local and pairwise conditional independencies which describe the relationships between the elements of the GMRF network [13]. These three types of conditional independency are equivalent and govern the factorization of the joint probability distribution [14]. In particular, the independence of two random variables 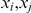 given the rest (*i.e.* the pairwise conditional independence) implies that the corresponding entry in the covariance inverse 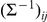 is zero, which indicates the non-existence of an edge between variables 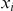 and 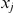 in the network. Consequently, pairwise conditional independence plays a fundamental role in network reconstruction since it provides information about the existence of edges between any pair of elements of the GMRF. Due to the symmetry of 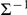, the inferred networks will be undirected. The graphical representation of a GMRF consists of an undirected graph that is defined as tuple 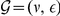 of a set of nodes 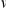 or genes in our case, and a set of edges, 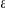, that describe the connections between the nodes or genes of the network.

In summary, estimating an undirected gene regulatory network graph is analogous to estimating the pairwise conditional independencies between the genes and, in our GMRF approach, is analogous to finding the zero entries of the inverse covariance matrix of the genes in the network. The covariance inverse 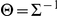 is also known as the precision matrix.

In a GMRF the conditional mean of 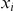 given the rest (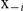) is linear in the measurements at the other nodes:
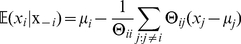
(2)


which has the same form as a multiple linear regression of 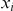 on 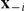 with regression coefficients 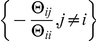 and depends only on the variables/nodes that are connected to 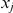.

### Graphical Lasso Algorithm

The sample covariance matrix 
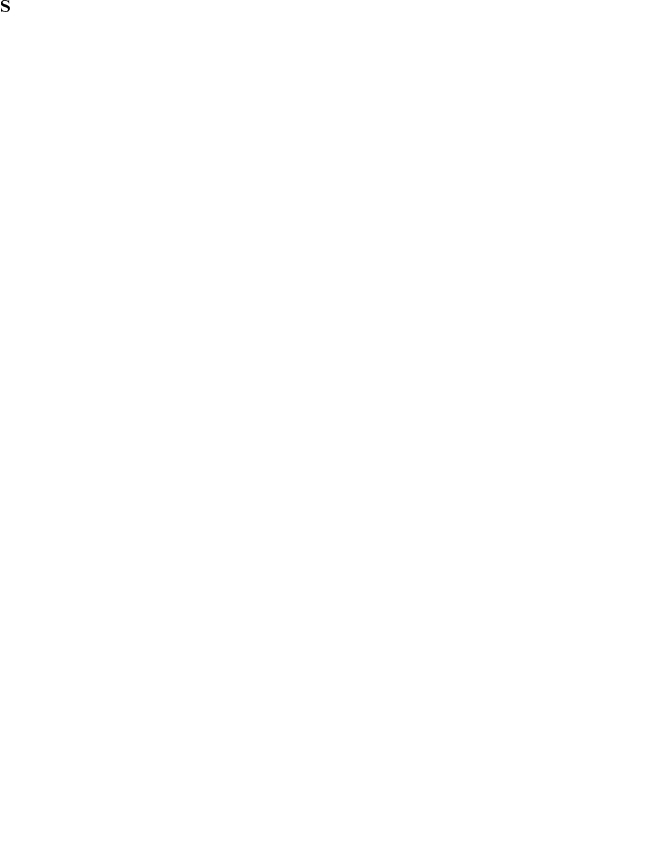
 of the gene expression measurements is a poor estimate of the true covariance matrix 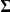 because of the low number of perturbations; its inverse, when it exists, will be dense. Equation (2) suggests that it is possible to learn about the dependence of 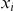 on 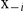 via (penalized) multiple linear regression [15], [16], in particular via the lasso [11] to obtain sparsity. However, things are a bit more complicated as 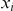 appears not only as response variable as in equation (2), but also as predictor in the equations for 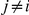.

**Figure 2:**
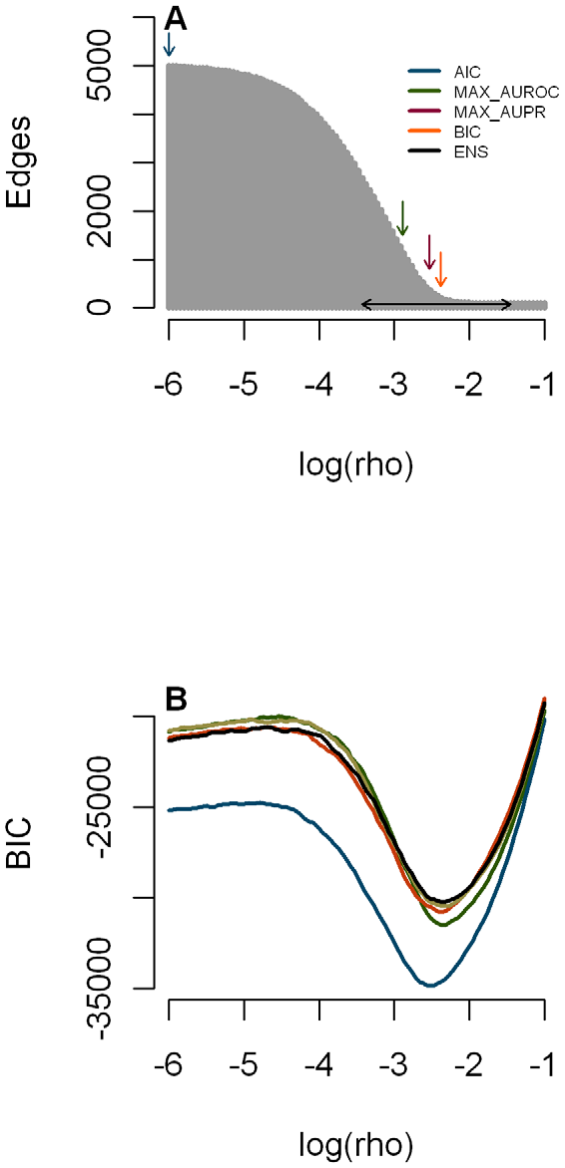
Influence of the graphical Lasso penalty on network complexity and Bayesian Information. A: Number of edges versus penalty for data set 3 in the multifactorial challenge with down arrows indicating the chosen 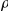 associated with (from left to right) AIC, MAX_AUROC, MAX_AUPR and BIC. The horizontal line connects 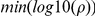 and 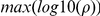 of the 50 best BIC networks chosen in the ensemble network. B: BIC versus penalty for the five data sets.

A both rigorous and efficient solution is the graphical lasso [9]. This maximizes the 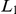 penalized loglikelihood 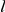 of the GMRF [10], defined by
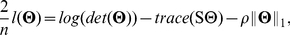
(3)with respect to the precision matrix 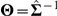. Here, 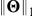 is the 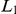 norm of 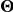, that is the sum of absolute values of the elements of 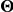, and 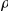 is a penalty that governs the sparsity of the network. In practice, this optimization problem is carried out for a series of 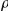 values, resulting in a series of networks that vary from very dense networks for low values for 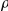 to very sparse networks for high values (Fig. 2A).

We now present a derivation of the graphical lasso algorithm [9], ending with an intuitive view of it. The gradient equation of the graphical lasso problem is [9], [10]

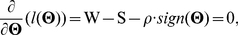
(4)where 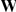 is the estimate of 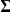, *i.e.* 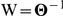. It is possible to solve this gradient equation (4), in an iterative block descendant fashion [10], by considering the partition of the GMRF 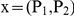 into the two groups 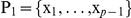 and 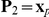. The corresponding partition of 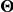 and its inverse 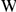 is
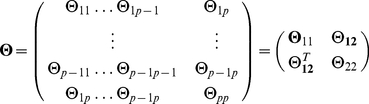
(5)

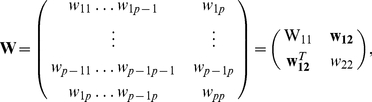
(6)where the column vector 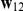 contains the marginal covariances between 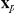 and the other elements in the GMRF 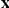. We partition 
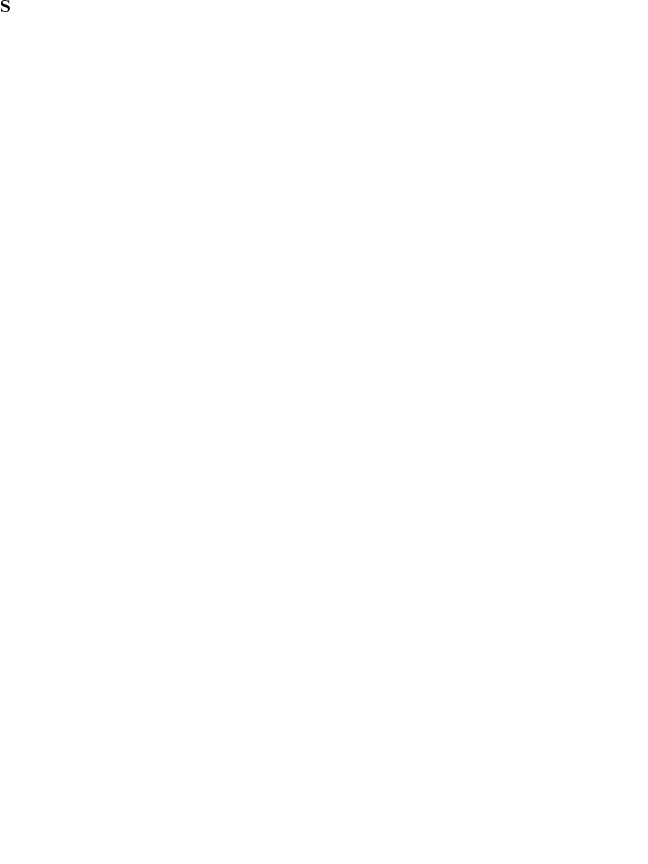
 correspondingly.

Friedman and coauthors [9] showed that for each given partition and 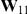, equation (4) can be solved for 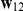 and 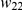 by a fast, regular lasso algorithm. The loglikelihood (3) is then maximized by considering all the possible partitions 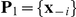, 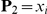 of the GMRF 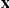 in turn and by iterating this process (Table 1). A key element is that, after 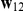 and 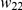 are calculated, they are inserted in the full 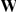 before a new partition is created. The matrix 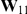, for a given partition, thus changes across iterations, until convergence.

**Table 1:** Graphical Lasso algorithm.

Graphical Lasso algorithm
**1**.Start with 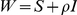 (diagonal is fixed from now)
**2**.Split matrix 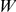 as in (6) taking in turn each variable to be the last column
**2.1** For each split solve equation (12) for 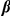 using the lasso coordinate descent algorithm
**2.2** Update 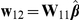
**2.3** In the final cycle, calculate for each split
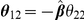 with 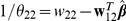
**3**.Repeat until convergence

We now show how, for a given partition, 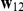, 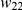 and the corresponding covariance inverse estimates can be obtained by a regular lasso algorithm. For a given partition, the partitioned version of equation (4) yields
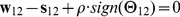
(7)


and also
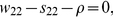
(8)


so that 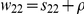. Equation (7) is less easy to solve as we do not know the sign of 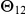 yet. But, as 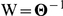, the partitioned version of
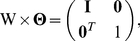
(9)


gives
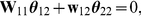
(10)


so that
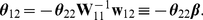
(11)


The sign of 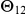 is thus opposite to the sign of 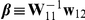 since 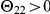. Rewriting equation (7) in terms of 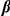 on using equation (11) gives
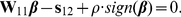
(12)


Equation (12) can be recognized as the gradient equation of the lasso problem [11]


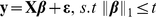
(13)

with 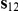 and 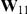 replacing 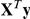 and 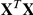, respectively. Each individual problem (12) is solved by coordinate descent [9], [17].

The graphical lasso algorithm (Table 1) can thus intuitively be viewed as a set of coupled lasso regression problems that share the same 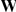 and 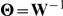. Table 1 summarizes the algorithm.

### Network selection

The efficiency of the Graphical Lasso algorithm allows to compute a great variety of network topologies just by evaluating a grid of penalty values 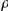. Since the parameter 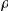 is responsible for the network sparsity, it is of particular interest to find which is the optimal estimated network in terms of this parameter. This problem of optimal network selection is equivalent to that of traditional model selection. We would want to have a model selection method that enables to select the adjacency matrix that best predicts the true topology. The main challenge here, is not only to discover the best network in terms of prediction accuracy, but to find a trade off between network sparsity and prediction accuracy in the hope to get closer to the true network. There are many model selection techniques. Cross validation [18], based on the performance of the estimated network into a test data set, is one of the most widely used. However, cross validation does not take into account the complexity of the selected network. With the goal in mind of finding sparse networks, we decided to minimize the Bayesian information criterion [19] and, just for the record, the Akaike criterion [20]

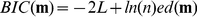
(14)

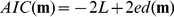
(15)


where 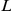 corresponds to the log-likelihood of model 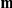 (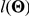 in equation (3) without the penalty term), 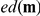 is the effective model dimension, here the number of non-zero edges in the network corresponding to model 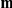 [21]–[23], and 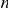 is the number of observations (perturbations). The BIC best enjoys the fame of being a trade off between model prediction and model complexity and thus to select sparser networks than those chosen by AIC or by cross validation. This is illustrated by the arrows in Fig. 2A showing the values of 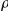 that minimize the BIC and AIC for data set 3.

In the submission to the DREAM4 challenge, each network had to consist of a ranked list of regulatory links ordered according to their confidence value. In the original description, links had to be directed but, as directionality is difficult to detect without experimental interventions, we consider here only undirected links. In our submission, we determined the confidence value of a link (edge) in a rather ad-hoc fashion as follows. We first set a series of 100 equispaced values in terms of 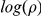 (Fig. 2). For each value of 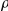, we then calculated the covariance inverse and associated BIC value. We then ordered the covariance inverses according to BIC, selected the 50 best ones and converted each to a network, *i.e.* adjacency matrix 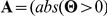), yielding 50 networks. The assigned confidence of an edge was the number of networks in which the edge was present divided by 50, the rationale being that we are more confident about an edge if it appears in more networks. As the resulting ranked network uses an ensemble of 50 networks, we term it the Ensemble network. In hindsight, we feel that the procedure is rather ad-hoc as it depends on the selected range of penalty values and the fraction of networks used in the ranking, which are both rather arbitrary. For that reason, we evaluated for this paper also the best BIC (and AIC) network per data set. For this evaluation we ordered the edges of the network according to the absolute value of their covariance inverse entry (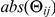), the rationale being that the size of 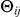 is traded against the loglikelihood of the network in equation (3) and thus has at least some statistical meaning. The network ranked in this way is termed the BIC (AIC) network. Selecting a particular 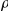 matters, because the models obtained via the graphical lasso algorithm for a grid of different penalties are not necessarily nested and therefore an edge that appears in a network with high 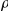 value can disappear when a smaller 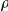 is considered. The corresponding entries of the covariance inverse may change non-monotonically with 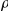. A referee suggested another ranking scheme, in which a given edge is ranked according to the maximal value of the penalty 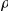 for which the putative edge is present in the predicted network. The network ranked in this way is termed MAX 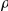.

### Post-hoc network validation

After submission, the true networks were released and it is thus possible to evaluate each submitted network according to the true one. Because of the confidence rating of edges, each submitted network is not just a single network but a ranked list of networks, containing from one to many edges, depending on the required confidence for an edge to exist and the total list size. For each given confidence threshold, the resulting network can be evaluated and compared with the golden standard, as follows.

Given two nodes in a network 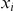 and 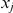, the edge prediction problem can have four possible outcomes when compared with the true network: (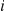) if the edge occurs in both the true and the predicted network, the prediction is called a *true positive* (TP), (
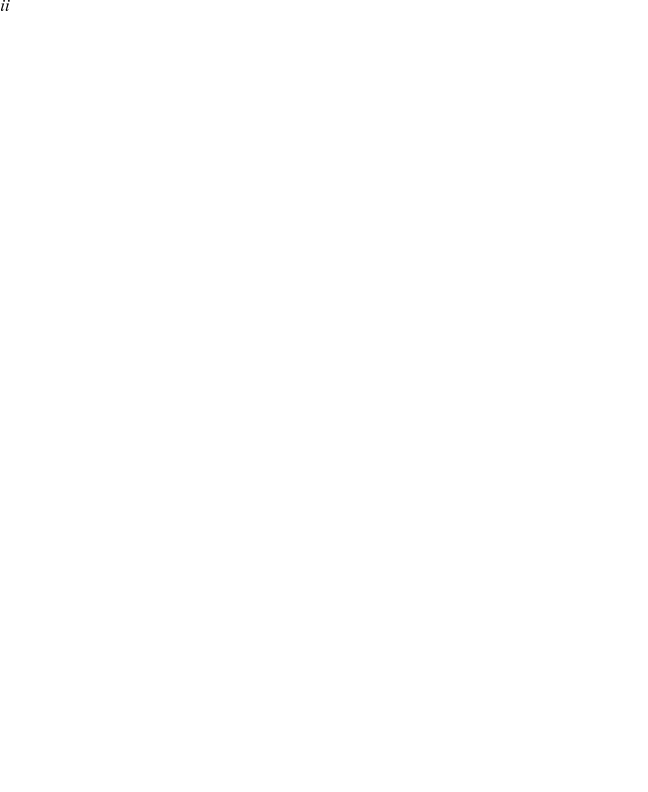
) if the edge is predicted but does not occur in the true network, it is a *false positive* (FP), (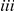) if the edge does neither occur in the true network nor in the predicted one, it is a *true negative* (TN), (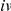) if the edge occurs in the true network, but is not predicted, it is a *false negative* (FN). Once the TP,TN,FN,FP events are counted, it is possible to calculate *True Positive Rate* (*TPR*) and *False Positive Rate* (*FPR*)
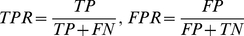
(16)


and *precision* and *recall* [24]–[26]

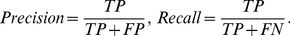
(17)


Precision is a measure of the exactness or fidelity of the network forecast, recall ( = TPR) is a measure of completeness, whereas FPR is the statistical Type I error (false alarm). In the words of [27], *“Precision may be defined as the probability that an object is relevant given that it is returned by the system, while the recall is the probability that a relevant object is returned”*.

**Figure 3:**
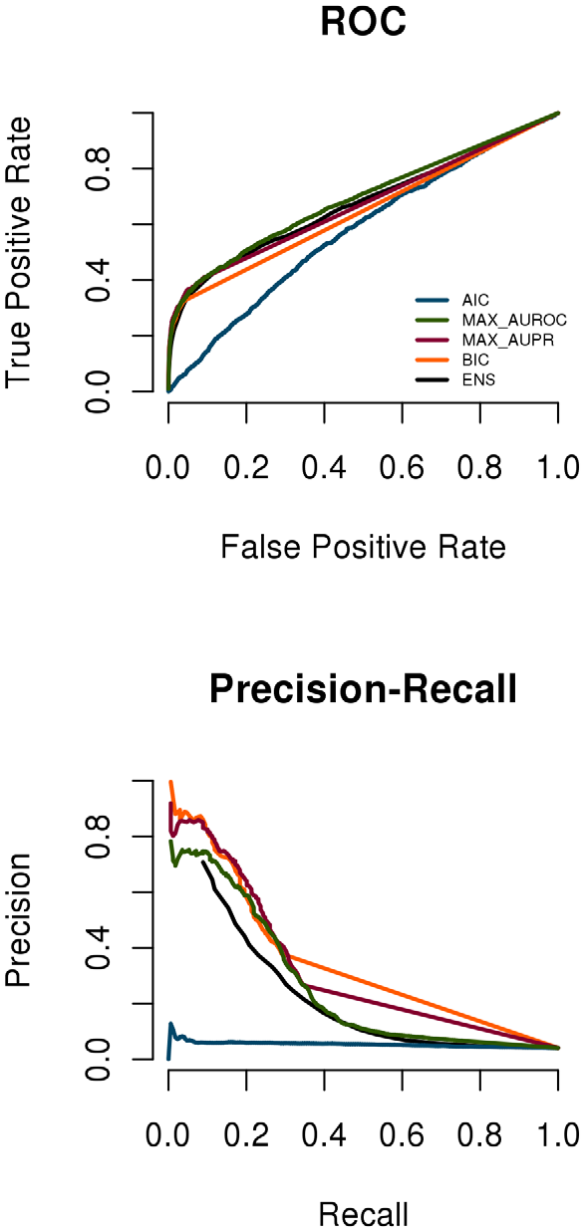
Performance of the five network reconstruction methods. The ROC and PR curves (Ensemble, AIC, BIC, MAX_AUPR and MAX_AUROC) are vertical averages of the curves for the five data sets.

By sliding the confidence threshold, the pairs (TPR, FPR) and (precision, recall) give rise to the Receiver Operating Characteristic (ROC) curve and Precision-Recall (PR) curve, respectively (Fig. 3). Popular overall measures of performance are then the Area Under the ROC and PR curves (AUROC and AUPR, respectively). The challenge organizers provided three more performance measures based on the P-value. The P-value is “the probability that a given or larger area under the curve value is obtained by random ordering of the T potential network links. Distributions for AUROC and AUPR were estimated from 100,000 instances of random network link permutations.” The overall P-value is then the geometric mean of the P-values of the individual data sets and the associated score is 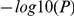. This score is calculated for both AUROC and AUPR and the two values are averaged to obtain the overall score.

We also investigated how well the graphical lasso could have done once we know the true networks. For this we determined the 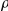 values maximizing AUROC and AUPR, yielding the AUROC and AUPR networks. The gap between the maximum AUROC and AUPR and those of the AIC, BIC and Ensemble networks indicates how much the results could have improved if we would have an ideal method of penalty selection.

## Results

The goal of the multifactorial sub-challenge was to reverse engineer five gene regulatory networks from training data consisting of steady-states levels of variation of the original networks, obtained after applying multifactorial perturbations to the system. The type of training data (only steady state, neither time series nor knockout/knockdown nor any other intervention data) motivated our choice for the GMRF approach to solve the problem in question.

The network topology was estimated by setting the edges to correspond to the nonzero elements of the estimated covariance inverse matrix 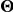. This covariance inverse was estimated from the training data by maximizing the penalized likelihood (3). The graphical lasso algorithm performed the computations in a very efficient and fast fashion, making it possible to compute the best covariance inverse for a series of 100 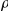 values within 60 seconds per data set on a laptop.

Fig. 2A shows for data set 3 how the number of nonzero elements in the covariance inverse decreases with increasing penalty 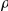 and also indicates the values of 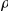 that minimize (from left to right) AIC, MAX-AUROC, MAX-AUPR and BIC. It also shows the range of the 50 best 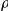 values used in the Ensemble network. As expected BIC yielded a much sparser network (ca. 500 edges) than AIC and Ensemble (both 5000 edges), whereas the true number of edges was 192 in network 3. The methods that select 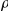 knowing the truth (MAX-AUROC and MAX-AUPR) produce networks that have both more edges than the network selected by BIC (Fig. 2A). Fig. 2B shows that 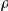 values minimizing BIC vary little across the five data sets.

Fig. 3 shows the ROC and PR curves for the different 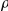 selection methods, averaged across the five data sets, while Table 2 shows the performance numerically. Fig. 3 and Table 2 show that AIC networks produced very poor results and that the Ensemble and MAX 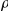 networks performed remarkably similar. BIC performed better than these in terms of AUPR score, but worse in terms of AUROC score. The network selected on the basis of maximum AUPR was better in terms of AUPR score, and about equal to Ensemble in terms of AUROC score. The network selected on the basis of maximum AUROC was only slightly better in terms of AUROC, and about equal to BIC in terms of AUPR score. The ensemble network that we submitted ranked fifth on the overall score. The modifications we investigated afterwards gave only modest improvement, as the different 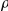 selection methods gave very similar results, except for the AIC method. Overall, BIC would have done slightly better, as it can be seen in Table 2. The winning team in the undirected 100 multifactorial sub-challenge had 37.3 and the team just above us had an overall score of 27.99. The worst score was close to 0.

**Table 2:** Average performance measures for different network reconstructions across data sets with standard deviations in parentheses.

Measures	Ensemble	AIC	BIC	MAX-AUPR	MAX-AUROC	MAX 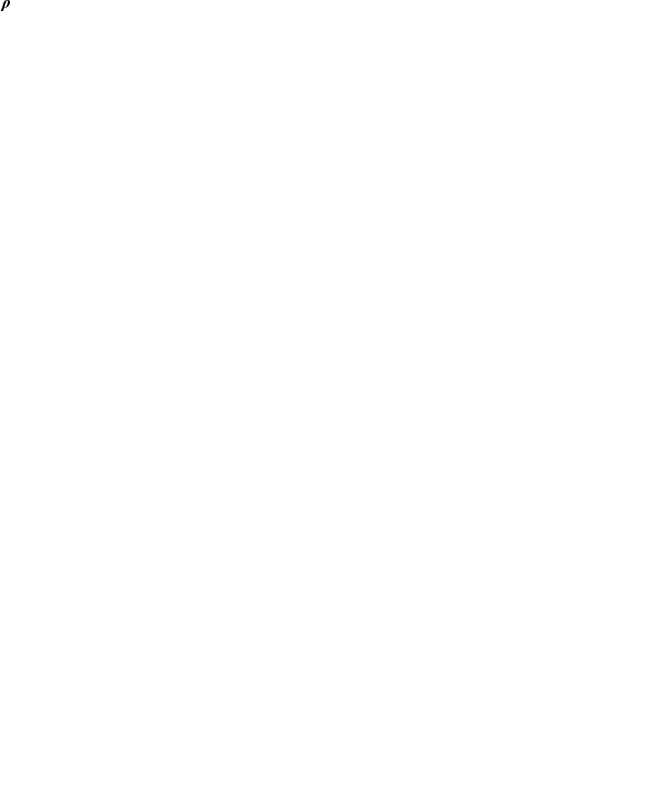
**AUPR**	0.23(0.04)	0.05(0.01)	0.26(0.06)	0.28(0.06)	0.26(0.08)	0.23(0.03)
**AUROC**	0.67(0.05)	0.58(0.02)	0.65(0.04)	0.68(0.04)	0.69(0.04)	0.68(0.04)
**Pr1Rec**	0.84(0.26)	0.18(0.27)	1(0.00)	0.82(0.25)	0.79(0.32)	0.81(0.27)
**Pr10Rec**	0.66(0.13)	0.06(0.02)	0.83(0.14)	0.83(0.13)	0.79(0.32)	0.65(0.13)
**Pr50Rec**	0.10(0.04)	0.05(0.01)	0.07(0.02)	0.10(0.04)	0.11(0.04)	0.11(0.04)
**Pr80Rec**	0.05(0.00)	0.04(0.01)	0.05(0.01)	0.05(0.01)	0.05(0.01)	0.05(0.01)
**AUPR score**	36.58	2.25	43.00	47.30	43.55	35.29
**AUROC score**	11.19	3.49	8.52	11.26	12.80	11.79
**Overall score**	23.89	2.87	25.76	29.28	28.17	23.54
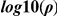	−	−6(0.00)	−2.38(0.08)	−2.60(0.07)	−2.94(0.34)	−

Pr1Rec, Pr10Rec, Pr50Rec, Pr80Rec represent precision at 1%, 10%, 50%, and 80% recall. The last row shows the best penalty value.

Furthermore, we studied the performance of the presented methodology with only half of the 100 perturbations. The results show for all the methods a decrease in the overall scores of about 20 percent (Table 3).

**Table 3:** Average performance measures for different network reconstructions across data sets, when only half of the perturbations were used, standard deviations in parentheses.

Measures	Ensemble	AIC	BIC	MAX-AUPR	MAX-AUROC	MAX 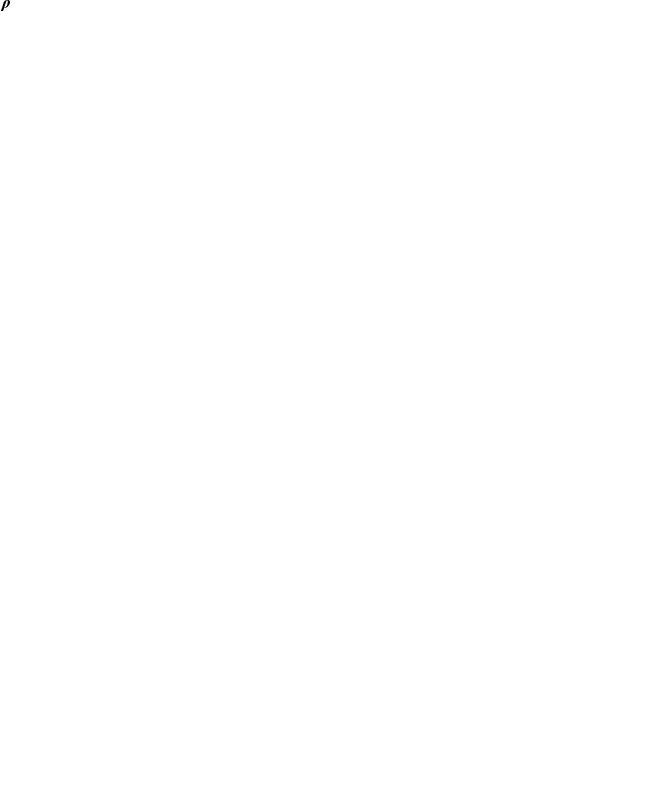
**AUPR**	0.18(0.04)	0.19(0.05)	0.21(0.05)	0.22(0.06)	0.22(0.06)	0.18(0.03)
**AUROC**	0.64(0.05)	0.63(0.04)	0.64(0.04)	0.63(0.04)	0.64(0.05)	0.64(0.05)
**Pr1Rec**	0.83(0.24)	0.82(0.26)	0.82(0.26)	0.92(0.18)	0.89(0.26)	0.83(0.24)
**Pr10Rec**	0.53(0.17)	0.64(0.23)	0.69(0.16)	0.73(0.14)	0.70(0.16)	0.53(0.16)
**Pr50Rec**	0.07(0.02)	0.07(0.02)	0.07(0.02)	0.07(0.02)	0.07(0.02)	0.07(0.02)
**Pr80Rec**	0.05(0.01)	0.04(0.01)	0.04(0.01)	0.04(0.01)	0.05(0.01)	0.05(0.01)
**AUPR score**	26.83	28.07	32.51	35.55	34.32	26.58
**AUROC score**	7.99	7.29	7.69	7.60	8.39	8.02
**Overal score**	17.41	17.68	20.10	21.57	21.36	17.30
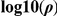	−	−3.00(0.00)	−2.72(0.27)	−2.41(0.15)	−2.66(0.23)	−

Pr1Rec, Pr10Rec, Pr50Rec, Pr80Rec represent precision at 1%, 10%, 50%, and 80% recall. The last row shows the best penalty value.

We also compared our approach with simple correlation networks, both for the full data (n = 100) and half the data (n = 50). Correlation networks were obtained by connecting two genes with an edge if the absolute value of their correlation was higher than a predefined threshold. The ranking of the edges was done according to the absolute value of the correlations. Table 4 shows that the performance depends somewhat on the threshold with the highest scores for threshold 0, that is, the relevance network (REL. in Table 4). The overall scores for the relevance networks (31.64 for n = 100, and 26.30 for n = 50) are higher than those obtained with the graphical lasso (Table 2 and Table 3). The overall score for 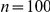 is equal to that of the second team in the multifactorial sub-challenge. The only two places were the graphical lasso wins over the relevance network are the precision at 1% and 10% recall for the full data. This advantage is then lost again for half the data.

**Table 4:** Average performance measures for different network computed using correlation reconstructions for thresholds 0 (**REL**), 0.4 and 0.8 with standard deviations in parentheses.

Measures	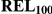	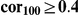	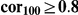	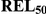	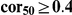	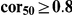
**AUPR**	0.26(0.06)	0.19(0.07)	0.05(0.02)	0.24(0.06)	0.20(0.06)	0.06(0.01)
**AUROC**	0.74(0.02)	0.61(0.04)	0.51(0.01)	0.70(0.04)	0.61(0.03)	0.51(0.00)
**Pr1Rec**	0.73(0.30)	0.73(0.30)	0.38(0.43)	0.84(0.36)	0.84(0.36)	0.84(0.36)
**Pr10Rec**	0.67(0.18)	0.67(0.17)	0.05(0.01)	0.73(0.15)	0.73(0.15)	0.05(0.01)
**Pr50Rec**	0.16(0.04)	0.06(0.01)	0.04(0.01)	0.11(0.03)	0.06(0.01)	0.04(0.01)
**Pr80Rec**	0.06(0.00)	0.04(0.01)	0.04(0.01)	0.05(0.01)	0.04(0.01)	0.04(0.01)
**AUPR score**	45.53	30.75	2.30	39.12	32.02	3.24
**AUROC score**	17.75	5.63	0.45	13.48	5.84	0.52
**Overall score**	31.64	18.19	1.38	26.30	18.93	1.88

Pr1Rec, Pr10Rec, Pr50Rec, Pr80Rec represent precision at 1%, 10%, 50%, and 80% recall when all (_100_) and only half of the perturbations (_50_) are considered.

## Discussion

We used a GMRF framework to tackle the problem of reverse engineering of regulatory networks based on data from random multifactorial perturbations, as posted in the DREAM4 challenge. The graphical lasso algorithm was used to compute the network topologies offering a very fast and easy computational set up, to provide a large range of candidate network topologies. This sub-challenge consisted of inferring directed networks, however, with the static nature of the provided training data, we believe that it is very complex to infer directionality or similarly causal relationships, and therefore we focused on the estimation of undirected networks which motivated the selection of our approach to tackle the problem.

We submitted networks with edge ranking based on edge frequency in an ensemble of the 50 best (out of 100) BIC networks. This ranked network turned out to perform very similar to MAX 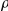 and similar to the single networks selected by BIC and ranked with confidence value proportional to the absolute value of the entry in the corresponding covariance inverse. The ensemble and MAX 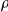 networks contained a larger number of edges in than the networks selected by BIC. Other ways to construct ensembles might perhaps improve the quality of the predictions and are a topic of further research.

The similarity between the ensemble and MAX 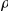 networks goes beyond their performance in Tables 2 and 3. The Spearman correlation between the confidence values, averaged across the five networks, was 0.97 and their ranked networks are therefore very similar. An explanation is that an edge that comes in early (at a high penalty value) gets high confidence in the MAX 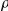 method, is likely to stay in the model for a large range of smaller 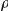 values and thus occurs in many of the networks we consider. Such an edge is likely to get a high rank in the ensemble method as well, because this method assigns the rank on the basis of the number of networks (among the 50 best BIC networks, see Fig. 2A) in which the edge occurs. Vice versa, a edge that comes in late (at a low penalty value, so that it gets low confidence in the MAX 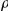 method) cannot occur in many of the 50 best networks and will thus receive low confidence in the ensemble method.

In our approach we assumed multivariate normality, that is normality of all marginal and conditional distributions of the measurements and, related to this, linearity between the conditional mean expression of a gene and the expression levels of its neighboring genes (equation (2)). These are strong assumptions, which are unlikely to hold true exactly. With few observations, these assumptions are hard to check. Q-Q plots, made assuming a sparse covariance inverse, did not show gross deviations from normality. A log-transformation of the measurements did not improve performance. The small data set size requires a simple model to produce reasonable results. Simplicity and speed are the key features of our approach.

Our study contributes to a better understanding of the properties and performance of the graphical lasso algorithm to estimate undirected networks. We showed that the method also works when the number of genes is larger than the number of perturbations. However, in this challenge relevance networks have shown a better performance, both for the full data and for half the data. For networks containing cliques that are locally dense, correlation networks might have an advantage compared to the sparsity imposed by the graphical Lasso algorithm with a single penalty term, as used in this study.
